# Perioperative PET/CT lymphoscintigraphy and fluorescent real-time imaging for sentinel lymph node mapping in early staged colon cancer

**DOI:** 10.1007/s00259-019-04284-w

**Published:** 2019-02-23

**Authors:** M. Ankersmit, O. S. Hoekstra, A. van Lingen, E. Bloemena, M. A. J. M. Jacobs, D. J. Vugts, H. J. Bonjer, G. A. M. S. van Dongen, W. J. H. J. Meijerink

**Affiliations:** 1 Department of Surgery, Cancer Centre Amsterdam, Amsterdam UMC, Vrije Universiteit Amsterdam, De Boelelaan, 1117 Amsterdam, The Netherlands; 2 Department of Radiology & Nuclear Medicine, Amsterdam UMC, Vrije Universiteit Amsterdam, Amsterdam, The Netherlands; 3 Department of Pathology Amsterdam UMC, Vrije Universiteit Amsterdam, Amsterdam, The Netherlands; 4 Department of Gastroenterology, Amsterdam UMC, Vrije Universiteit Amsterdam, Amsterdam, The Netherlands; 5 Department of Operation Rooms and MITeC Technology Center, Radboud University Medical Centre, Nijmegen, The Netherlands

**Keywords:** Colon cancer, Sentinel lymph node, PET/CT imaging, Near-infrared imaging, Fluorescence

## Abstract

**Purpose:**

Using current optical imaging techniques and gamma imaging modalities, perioperative sentinel lymph node (SLN) identification in colon cancer can be difficult when the SLN is located near the primary tumour or beneath a thick layer of (fat) tissue. Sentinel lymph node mapping using PET/CT lymphoscintigraphy combined with real-time visualization of the SLN using near-infrared imaging has shown promising results in several types of cancer and may facilitate the successful identification of the number and location of the SLN in early colon cancer.

**Methods:**

Clinical feasibility of PET/CT lymphoscintigraphy using preoperative endoscopically injected [^89^Zr]Zr-Nanocoll and intraoperative injection of the near-infrared (NIR) tracer Indocyanine Green (ICG) was evaluated in ten early colon cancer patients. Three preoperative PET/CT scans and an additional ex vivo scan of the specimen were performed after submucosal injection of [^89^Zr]Zr-Nanocoll. All SLNs and other lymph nodes underwent extensive pathological examination for metastases. A histopathological proven lymph node visible at preoperative PET/CT and identified at PET/CT of the specimen was defined as SLN.

**Results:**

A total of 27 SLNs were harvested in seven out of eight patients with successful injection of both tracers. In one patient no SLNs were assigned preoperatively. In two patients injection of [^89^Zr]Zr-Nanocoll failed due to incorrect needle positioning. Twenty-one (78%) SLNs were found intraoperatively using NIR-imaging. Eleven of the 27 (41%) SLNs were located near the primary tumour (< 2 cm). Those six SLNs not found intraoperatively with NIR-imaging were all located close to the tumour. In all seven patients at least one SLN could be assigned at preoperative imaging 24 h after tracer administration. One SLN contained metastases detected by immunohistochemistry. No metastases were found in the non-SLNs.

**Conclusions:**

This study shows the potential of preoperative PET/CT lymphoscintigraphy to inform the surgeon about the number and location of SLNs in patients with early colon cancer. The additional use of NIR-imaging allows for intraoperative identification of these SLNs which are invisible with conventional white light imaging. Further research is necessary to improve and simplify the technique. We recommend perioperative SLN identification using a preoperative lymphoscintigraphy scan just before surgery approximately 24 h after injection. Additionally a postoperative scan of the specimen combined with intraoperative real-time NIR-imaging should be performed.

**Electronic supplementary material:**

The online version of this article (10.1007/s00259-019-04284-w) contains supplementary material, which is available to authorized users.

## Introduction

Colorectal cancer (CRC) is the second most common malignancy in the Western world and the fourth leading cancer-related cause of death worldwide [1]. Lymph node involvement is still the strongest prognostic factor and serves as the most important selection criterion for adjuvant chemotherapy [2]. The introduction of CRC screening programs will increase the number of early staged CRC (T1-T2 disease) [3, 4]. The low risk of lymph node metastases in these early staged tumours makes local excision of the primary tumour an attractive treatment option [5]. However, uncertainty regarding undetected lymph node metastases makes current treatment of segmental resection with en-bloc resection of lymph nodes unavoidable in early CRC. Despite complete surgical resection, up to 20 to 30% of patients with early CRC show disease recurrence and eventually die within five years of initial treatment [6, 7]. This high recurrence rate in node negative patients is probably the result of understaging due to missed metastases in lymph nodes during routine histopathological examination [8–10]. Conversely, the majority of patients with true negative lymph nodes are exposed to unnecessary surgery-related morbidity and mortality at rates of up to 13.5 and 2.0%, respectively [11]. Sentinel lymph node (SLN) identification could offer a solution by detecting the lymph nodes with the most direct drainage from the primary tumour and therefore with the greatest chance of harbouring metastases.

In melanoma and breast cancer, SLN biopsies are routinely performed using a combination of preoperative colloid SPECT lymphoscintigraphy and perioperative guidance by gamma-probe and preoperatively injected blue dye. The combination of these techniques allows for preoperative identification of the number and location of the SLNs, and real-time identification of the SLNs versus surrounding fat [12–15]. SLN identification in colon cancer seems more challenging than in breast cancer and melanoma. Firstly, SLNs of the colon are more often smaller (˂ 1 cm), located beneath a thick layer of (fat) tissue and not visible with conventional white light imaging. Secondly, number and location of SLNs of colon carcinoma are less predictable. Additionally, it seems that more than one node can be assigned as SLN frequently and they appear to be often located near the tumour. An additional difficulty of SLN identification in colon cancer is the absence of the intraoperative sense of touch since laparoscopy is the preferred surgical approach for colon resections. These drawbacks underline the need for a high quality optic tracer. The limited resolution of planar or SPECT scintigraphy may preclude proper preoperative SLN identification due to the shine-through effect from the tracer depot [16, 17]. Intraoperative identification is difficult because blue dye cannot be seen through fatty tissue and its relatively small particle size causes rapid passage through lymphatic channels, limiting its usefulness in detecting the earliest tumour draining lymph nodes [18, 19].

Near-infrared fluorescent tracers, and especially Indocyanine Green (ICG), exhibit more favourable characteristics for intraoperative detection of SLNs compared to blue dye (e.g. larger particle size and real-time, high/resolution optical guidance) [20]. PET scanners have a better spatial resolution than conventional gamma imaging modalities, and allow for dynamic 3D imaging. In oral cancer patients, [^89^Zr]Zr-Nanocoll PET/CT provided detailed anatomical localization of tracer-foci and potentially improved identification of the SLNs even when they were located near the injection spot [21]*.* In colorectal cancer preoperative surgical planning using PET/CT combined with real-time NIR-staining of the SLNs using ICG might offer a solution for successful SLN biopsy.

The main purpose of this study was to establish if preoperative [^89^Zr]Zr-Nanocoll PET/CT imaging is a useful technique to identify the number and location of SLNs in early colon cancer. Concordance and accuracy between number, location and histopathological outcomes of preoperative and postoperative assigned SLNs at PET/CT imaging and intraoperative identified SLNs using real-time fluorescent NIR-imaging are determined.

Secondly, we aimed to investigate the pharmacokinetics of [^89^Zr]Zr-Nanocoll to optimize the logistics of SLN biopsies involving radiolabelled nanocolloid.

## Materials and methods

### Patients

Patients were eligible if at least 18 years of age and scheduled for a laparoscopic resection of a histopathologically proven colon carcinoma or suspected malignant lesion seen during colonoscopy. Oral and written consent was mandatory for inclusion.

Exclusion criteria were reduced physical condition (ASA IV), suspected or proven lymph node involvement or distant metastases seen on routine preoperative imaging (CT-scan), a tumour too large to pass endoscopically, claustrophobia and allergy for iodine. The Medical Ethics Committee of the Amsterdam UMC–Vrije Universiteit Amsterdam and the National Competent Authority approved the study. The study is registered in the Clinical Trial database (NCT02850783).

### Study design

**Fig. 1:**
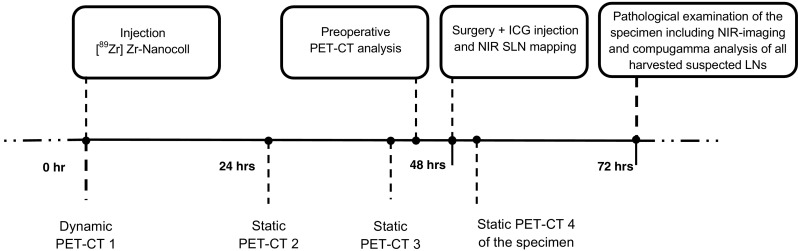
Time schedule of the study protocol

**Fig. 2:**
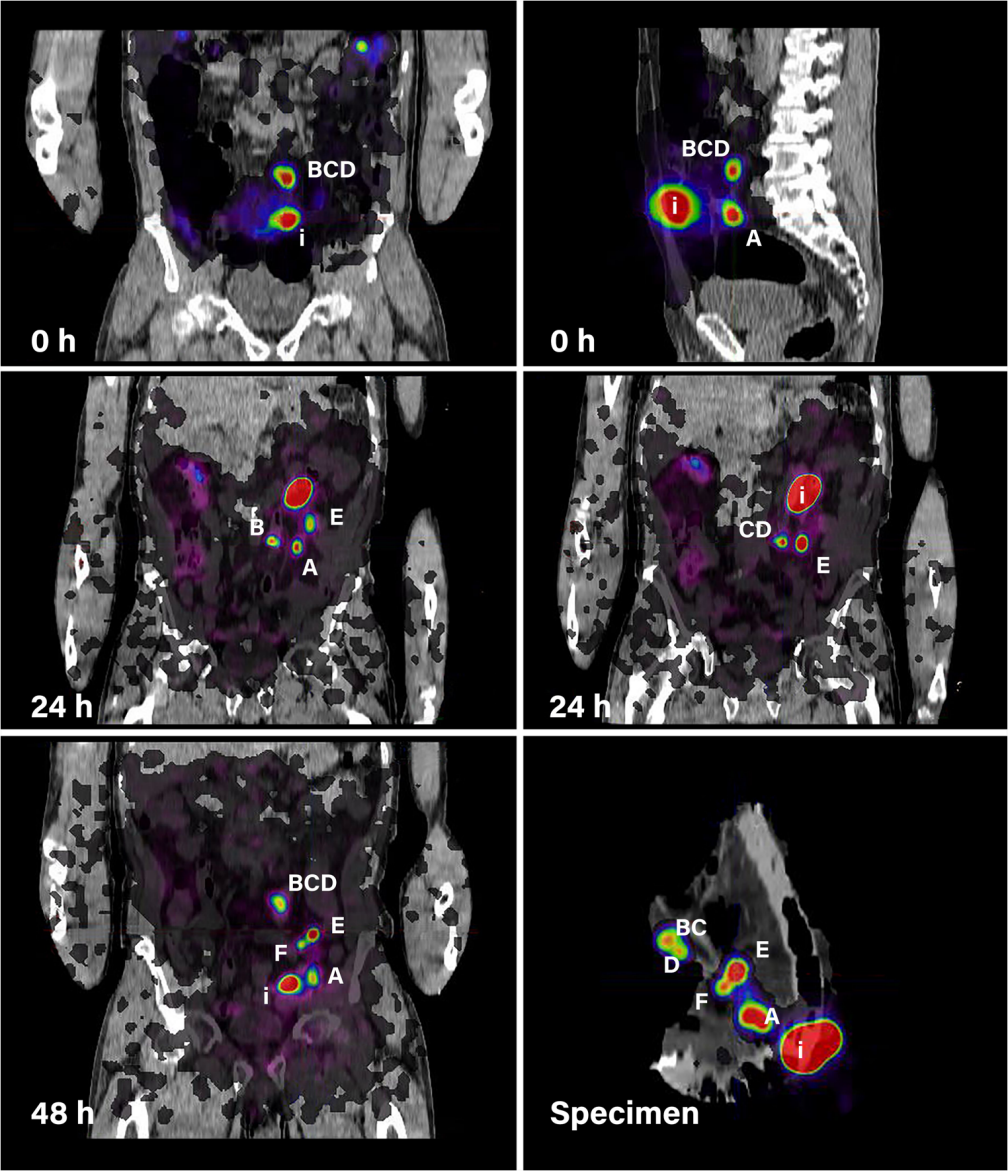
SLN identification at PET/CT images of patient 2. Injection side is shown as `i` in all images. Foci A, B, C and D showed in total five sentinel lymph nodes and were already apparent at first scan (0 h after injection). Foci E represented two sentinel lymph nodes first seen at the second PET/CT scan (24 h after injection) and F was first seen at the third scan after 48 h. Lymph nodes E and F were probably hidden behind the other 'hotter' nodes. All SLNs were intraoperatively identified using near-infrared imaging

The study protocol (Fig. 1) consisted of three preoperative PET/CT scans after injection of 0.4 mL median 2.12 (1.69–2.85) MBq [^89^Zr]Zr-Nanocoll approximately 46 (43–48) hrs before surgery and intraoperative injection of ICG. Detailed information concerning the injection technique can be found in the supplementary material (Supplementary material 1). The PET/CT scan nr. 1 (Ingenuity; Philips Healthcare) consisted of 3–5 dynamic frames of 5 min each and started circa one hour after tracer injection. Two static PET/CT images were made one day (scan nr. 2) and two days (scan nr. 3) after tracer administration (3–5 frames, 5 min each), respectively. Before surgery the results of the PET/CT images were compared with respect to the total number, localization and intensity of possible SLNs by a senior nuclear medicine physician (O.S.H). Results were discussed with the operating surgeon. A SLN was defined when focal tracer accumulation was evident in the mesocolon. At start of surgery after general anaesthesia, a second colonoscopy served to inject the fluorescent tracer Indocyanine Green (ICG) at the base of the tumour using one single injection. During surgery, a NIR laparoscopic device was used (Olympus; Tokyo, Japan). The SLNs were detected by means of fluorescence and the 'hot spot' locations preoperatively assigned at PET/CT. Intraoperative fluorescent SLNs were marked with a suture. All patients underwent conventional oncological laparoscopic resection after SLN(s) marking with a suture. A fourth PET/CT scan (scan nr. 4) of the surgical specimen was made directly after surgery to confirm correct identification of preoperatively identified 'hot spots' and to facilitate comparison of PET/CT imaging and pathological findings (Fig. 2). After identification and additional suturing of radioactive lymph nodes found at PET/CT or with NIR- imaging, the specimen was transferred to the Department of Pathology for examination of the specimen including all lymph nodes.

### [^89^Zr]Zr-Nanocoll

[^89^Zr]Zr-Nanocoll is the PET corollary of ^99m^Tc-Nanocoll used for SPECT scintigraphy. Heuveling et al. [22] showed that Nanocoll pharmacokinetics are independent of the radiolabel (either ^89^Zr or ^99m^Tc) and the 78 h physical half-life of ^89^Zr-Nanocoll allows for flexibility in SLN observation time. [^89^Zr]Zr-NCS-Bz-DFO-Nanocoll (hereafter called [^89^Zr]Zr-Nanocoll) was produced according to the previously reported method in Heuveling et al. [22] under good manufacturing practice compliant conditions. [^89^Zr]Zr-Nanocoll was filter sterilized which resulted in a sterile final product with less than 2.5 endotoxin units/mL. The radiochemical purity was >99.9%.

### Histopathology

Examination of the specimen by the pathologist followed after fixation of the specimen in formalin for at least 24 h. After fixation the pathologist harvested the perioperative suture-marked structures assigned as SLNs first and stored them separately. Thereafter, the pathologist searched for additional lymph nodes by palpation and slicing of the whole specimen. Round, smooth and rigid structures similar to lymph nodes were harvested and stored separately too. Location of each potential SLN and all non-SLNs were compared with the locations assigned at PET/CT imaging. All lymph nodes were reassessed for fluorescence with the NIR-laparoscope and gamma well counter to reveal radioactivity.

All harvested SLNs and non-SLNs were bisected along the longest axis, paraffin embedded and stained with haematoxylin & eosin (H&E). Individual SLNs were embedded separately. If the lymph nodes were negative after routine H&E staining, all nodes were sectioned (3–4 μm thick) at 150 μm intervals and examined at three levels with H&E-staining and immunohistochemistry with the epithelial marker CEA (Clone 1117; DAKO Netherlands M7072), CAM 5.2 (3,455,799; BD Biosciences Netherlands) and CK19 (M0888, clone RCK 108; DAKO The Netherlands). Metastases between 0.2 mm and 2.0 mm were classified as micrometastases, and metastases smaller than 0.2 mm as isolated tumour cells according to the TNM 5 classification.

### Image analysis

All PET/CT scans including the PET/CT of the specimen, were postoperatively reanalyzed and the results compared with respect to the total number and location of foci by a nuclear medicine physician (O.S.H) who was blinded to surgical findings and pathology results.

The location of all lymph nodes found by the pathologist were compared with pre-, and postoperatively assigned SLNs at PET/CT imaging using Vinci software (Vinci 2.36.0; Max-Planck-Institut fur Neurologische Forschung, Cologne, Germany). Volumes of interest (VOI) were used to delineate the amount of radioactivity for pharmacokinetics [23].

### Definition of sentinel lymph nodes

A histopathological proven lymph node visible at preoperative PET/CT and identified at PET/CT of the specimen was classified as SLN. Lymph nodes only stained by ICG or unstained nodes identified by the pathologist were defined as other lymph nodes.

### Statistical analysis

Data were analysed using SPSS software (SPSS version 22.0; SPSS, Inc.; Chicago, IL). Point estimates and distribution were expressed as median and range, respectively. The Mann-Whitney U test was performed to determine the statistical significance of continuous variables. Results were considered as statistically significant at a *P* level of less than 0.05.

## Results

We included ten patients with early colon cancer, and patient characteristics are shown in Table 1. None of the patients experienced any adverse reaction following [^89^Zr]Zr-Nanocoll administration. In one patient (Table 2, patient 4, foci A) an SLN with isolated tumour cells was found. This SLN was detected with both imaging modalities and located >2 cm from the primary tumour. No metastases were found in other SLNs or lymph nodes in the remaining patients. ICG injection was successful in all patients, but [^89^Zr]Zr-Nanocoll administration failed in two cases (Table 1; patients 3 and 4). In one patient tracer had been injected through the subserosal colonic layer in the abdominal cavity (patient 3). In another patient injection failed due to needle luxation outside the tumour during [^89^Zr]Zr-Nanocoll administration (patient 4). Both resulted in low uptake of radioactivity in the primary tumour and high background radiation due to spill in the abdominal cavity or colonic lumen. Both patients were excluded from further analysis.

**Table 1:** Basic patient characteristics

Patient	Gender	Age (years)	BMI (kg/m^2^)	ASA I-III	Tumour side	Tumour size (cm)	T-stage	N-stage
1	Male	74	24.1	II	Sigmoid	0.5	1	N0
2	Male	67	23.0	II	Sigmoid	2.8	1	N0
3	Male	65	21.9	II	Flexura hepatica	4.8	3	N0
4	Male	75	36.1	III	Flexura lienalis	1.0	3	N1
5	Female	65	27.4	II	Cecum	3.4	2	N0
6	Male	77	23.8	II	Sigmoid	3.0	2	N0
7	Male	74	23.3	II	Colon ascendens	3.2	1	N0
8	Male	76	26.6	II	Sigmoid	3.9	2	N0
9	Male	69	27.8	I	Sigmoid	2.5	2	N0
10	Female	63	22.6	II	Flexura lienalis	4.0	2	N0
Total		71.5 (63–77)	24.0 (21.9–36.1)			3.1 (0.5–4.8)

**Table 2:** Results of sentinel lymph node identification with PET/CT and near-infrared imaging for each patient

Patient	Foci	LN status after pathology	Assigned as SLN by PET-CT imaging before surgery	Marked intraoperatively using NIR-ICG	Additional ex vivo positive for NIR-ICG	Marked postoperatively at PET/CT of the specimen	Identified at postoperative analysis of the 1st PET/CT	Identified at postoperative analysis of the 2nd PET/CT	Identified at postoperative analysis of the 3th PET/CT
1	A	No LN tissue	Yes	Yes		Yes	–	–	Yes
	B	No LN tissue	–	Yes		–	–	–	–
	C	No LN tissue	–	Yes		–	–	–	–
	D	No LN tissue	–	Yes		–	–	–	–
	E	No LN tissue	–	–	Yes	Yes	–	–	–
	F	LN	–	Yes		Yes	–	–	–
	LN diaphragm	Not harvested	Not harvested				–	Yes	Yes
	LN pre-aortal 7 x not found	Not harvested	Not harvested				–	Yes	Yes
							1x not found	4 x not found	2 x not found
2	A	2 x SLN	Yes	Yes		Yes	Yes	Yes	Yes
	B	SLN	Yes	Yes		Yes	Yes	Yes	Yes
	C	SLN	Yes	Yes		Yes	Yes	Yes	Yes
	D	SLN	Yes	Yes		Yes	Yes	Yes	Yes
	E	2 x SLN	Yes	Yes		Yes	–	Yes	Yes
	F	SLN	Yes	Yes		Yes	–	–	Yes
3	A	SLN	Yes	Yes		Yes	–	Yes	Yes
	B	SLN	Yes	Yes		Yes	–	Yes	Yes
	C	LN	–	Yes			–	–	–
	D	LN	–	Yes			–	–	–
	1 x not found								1 x not found
4	A	SLN ITC	Yes	Yes		Yes	–	Yes	Yes
	B	(IHC)	Yes	Yes		Yes	–	–	Yes
	C	SLN	Yes	Yes		Yes	–	Yes	–
	D	SLN	–	Yes		Yes	–	–	–
	E	LN	–	Yes		–	–	–	–
	F	LN	–	Yes		Yes	–	–	–
	G	LN	–	–	Yes	Yes	–	–	–
	5 x not found	LN	–					1x not found	4x not found
5	A	2 x SLN	Yes	Yes		Yes	Yes	Yes	–
	B	SLN	Yes	–	Yes	Yes	–	–	Yes
	C	LN	–	Yes		Yes	–	–	–
	D	LN	–	–	Yes	Yes	–	–	–
	E	LN	–	Yes		Yes	–	–	–
	F	2 x LN	–	Yes		Yes	–	–	–
	3 x not found		–				1x not found		2 x not found
6	A	SLN	Yes	Yes		Yes	Yes	Yes	Yes
	B	SLN	Yes	Yes		Yes	Yes	–	Yes
	C	LN	–	Yes		Yes	–	–	–
	D	LN	–	Yes		–	–	–	–
	E	LN	–	Yes		–	–	–	–
	F	LN	–	Yes		–	–	–	–
	5 x not found		–	–		–	2 x not found	1 x not found	2 x not found
	1 x no LN tissue		–	1 x no LN tissue			–	–	–
7	A	2 x SLN	Yes	Yes		Yes	–	Yes	Yes
	B	SLN	Yes	Yes		Yes	Yes	Yes	Yes
	C	SLN	Yes	–	Yes	Yes	–	Yes	Yes
	1 x not found						1 x not found		
8	A	SLN	Yes	Yes		Yes	–	Yes	Yes
	B	SLN	Yes	–	Yes	Yes	Yes	Yes	Yes
	C	3 x SLN	Yes	–	Yes	Yes	–	Yes	Yes
	D	LN	–	Yes		Yes	–	–	–
	E	LN	–	–	Yes	Yes	–	–	–
	F	LN	–	Yes		Yes	–	–	–
	G	LN	–	–	Yes	Yes	–	–	–
	1 x not found		–						1 x not found
Total	71 foci			21 x SLN	6 x SLN	27 x SLN	11 x SLN	23 x SLN	24 x SLN
	21 foci with 27 SLN			16 LN	4x LN	14 x LN			
	19 foci with 20 LN								
	23 not found								
	6 no LN tissue								
	2 not harvested								

*LN* = Lymph Node; SLN = Sentinel Lymph Node; NIR-ICG = Near infrared-Indocyanine Green

At preoperative PET/CT imaging we assigned 24 potential SLN foci (Table 2). One subdiaphragmatic and one preaortic potential lymph node (Table 2, patient 1) were not harvested because they were located too far from the resection margins, which would hamper the conventional resection. Another focus was perioperative found but did not contain lymphatic tissue (Table 2, patient 1, focus A). In this patient no other SLNs were assigned preoperatively. After exclusion of these three foci, a final number of 21 foci in seven out of eight patients with successful injection of both tracers were identified. Some foci proved to contain more than one SLN, so that these 21 foci compromised 27 true SLNs. All 21 foci were preoperatively assigned by the nuclear medicine consultant just before surgery. All 27 SLNs revealed fluorescence of which 21 (78%) were detected intraoperatively using NIR-imaging. Eleven of the 27 (41%) SLNs were located near the primary tumour (< 2 cm). Those six SLNs not found intraoperatively with NIR-imaging were all located close to the tumour.

An additional 14 lymph nodes were identified at PET/CT imaging of the specimen. All revealed fluorescence of which ten were identified by intraoperative NIR-imaging. Six out of these 14 lymph nodes were located near the primary tumour; three were only visible at PET/CT of the specimen and another three with both imaging modalities. Another ten foci were only intraoperatively identified with NIR imaging. Four of these fluorescent foci contained fat tissue (patient 1; foci B, C, D, E). The remaining foci revealed six lymph nodes all of which were located far from the primary tumour.

All specimens were submitted for pathological examination. An additional 172 suspected lymph nodes were harvested from the specimen by the pathologist. Histopathological examination showed 102 true lymph nodes. Fat or blood vessels were found at 70 foci of which 15 showed fluorescence probably as a result of dye leakage after disruption of lymphatic vessels during specimen extraction.

Of the 102 true lymph nodes, 41 nodes (40%) showed fluorescence with radioactivity counts of 14.3 10^−3^[0.36–13.3^−3^] MBq/node and 0.97 [0.028–9.1] %ID/node, which was significantly lower than the SLN radioactivity; 68.3 × 10^−3^[4.7–215.9^−3^] MBq/node and 4.7 [0.32–13.2] %ID/node, (*p* = 0.0001). Mean radioactivity of lymph nodes without uptake of ICG was 1.2 × 10^−3^ (0.33–8.3 × 10^−3^) MBq and 0.08 (0.03–0.55) %ID/node, which was significantly less than activity in the SLNs and 41 fluorescent lymph nodes (both *p* = 0.0001). None of the lymph nodes additionally found in the specimen showed metastases.

### Pharmacokinetics and biodistribution

**Fig. 3:**
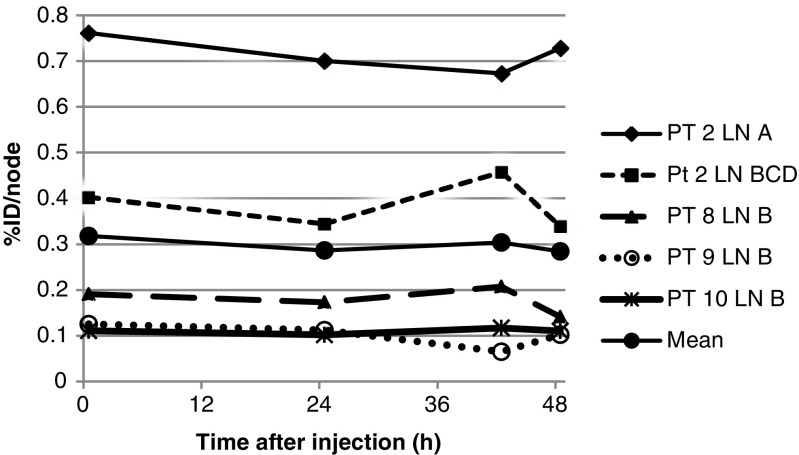
Lymph node kinetics of [^89^Zr]Zr-Nanocoll

**Fig. 4:**
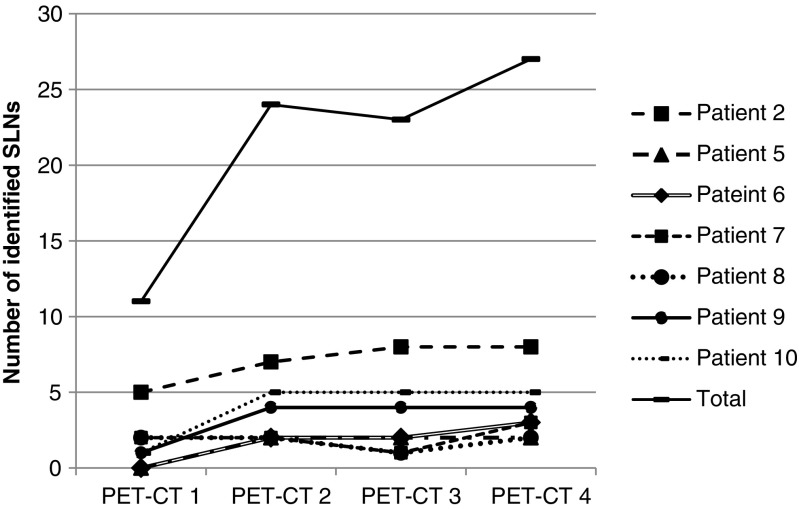
Sentinel lymph node identification by PET/CT imaging over time

The pharmacokinetics of [^89^Zr]Zr-Nanocoll are shown in Fig. 3. Pharmacokinetics were calculated using the amount of radioactivity in seven out of the eight SLNs identified at all PET/CT scans. Exclusion of one SLN occurred since no reliable VOI be drawn due to its location near (< 2 cm) the tumour. PET/CT scans were made 0.8 (0.17–1.37) hrs, 24.18 (16.07–26.62) hrs, 42.02 (40.08–42.33) hrs and 48.34 (46.2–5.25) hrs after injection, respectively. Not all SLNs were seen at all three preoperative PET/CT scans. Eleven SLNs were visible at the first PET/CT scan. At the second PET/CT scan 23 SLNs were seen. One SLN seen at the first PET/CT was not visible at the second scan and 13 were first identified at the second. Twenty-four SLNs were marked at the third PET/CT just before surgery. Three SLNs seen at the second PET/CT scan were not visible at the third scan. Another three SLNs were first identified at this time-point. (Fig. 4).

## Discussion

In the present study we demonstrated the feasibility of PET/CT lymphoscintigraphy combined with optical real-time NIR-imaging using [^89^Zr]Zr-Nanocoll and ICG to identify the SLNs in early stage colon cancer patients. Perioperative SLN identification succeeded in seven out of eight patients in which a median number of three SLNs were found. All SLNs revealed radioactivity and fluorescence, but six SLNs were not identified with NIR-imaging intraoperatively. These six lymph nodes were all located near (< 2 cm) the primary tumour and were probably covered due to the shine-through effects from the injection depot. When attempting to identify SLNs near the tumour, our results demonstrate that PET/CT imaging is a reliable technique, although PET/CT of the surgical specimen may improve the accuracy. Overall, these findings suggest that PET/CT lymphoscintigraphy combined with NIR-imaging could be a useful method for SLN identification in patients with early-staged colon cancer. The preoperative PET/CT images could guide the surgeon to the number and location of SLNs which are currently unknown for colon cancer. Identification of these SLNs with NIR-imaging allows for intraoperative detection of these SLNs which is not possible with conventional white light imaging since SLNs are not visible with the naked eye.

To the best of our knowledge, this is the first study using [^89^Zr]Zr-Nanocoll radiocolloid and PET/CT with NIR-imaging as SLN mapping technique in colon cancer. The majority of studies in the literature used SPECT/CT alone or in combination with an optical tracer, typically blue dye. The limited resolution of gamma-cameras combined with the restricted visibility of blue dye through skin and fatty tissue and its rapid distribution through lymphatic channels, makes it difficult to detect SLNs in colon cancer, in particular since lymphatic drainage patterns, location and number of SLNs are unknown and unpredictable. The biodistribution data of [^89^Zr]Zr-Nanocoll over time presented here, combined with the extended pathological examination of the specimen, provided essential anatomical information on lymphatic drainage patterns of the primary tumour towards SLNs.

The SLN procedure is based on the concept that tumour metastases occur in an orderly and sequential manner. The SLN(s) is/are the first lymph node(s) that receives lymphatic drainage directly from the primary tumour and therefore has the highest probability of harbouring metastases [24]. Based on this theory, we hypothesized that the lymph nodes with the highest radioactive counts ('hottest' nodes) are most likely the first draining nodes from the primary tumour and therefore can be considered as SLNs. These 'hottest' nodes are by definition the nodes visible at PET/CT imaging. Therefore we classified a node as SLN when it was a histopathological proven lymph node visible at preoperative imaging and identified at PET/CT of the specimen. Similarly to breast cancer and melanoma, more than one 'hot' node were assigned as SLNs in all patients. This phenomenon could be attributed to passing of the tracer through the actual SLN into other nodes or due to divergent drainage patterns from the primary tumour [25–27]. Not all identified SLNs were visible at PET/CT imaging at all time points. Moreover, 14 lymph nodes were first seen at PET/CT of the specimen and therefore not classified as SLN. Both could be the result of physical bowel movements which changes the anatomical position of that part of the colon containing the injection depot and precludes SLN detection, as visualized in Fig. 2. However, it can be argued that lymph nodes first seen at postoperative PET/CT should also be considered as SLNs, especially when these lymph nodes are located near the primary tumour. For accurate SLN identification we therefore recommend combined preoperative and postoperative PET/CT. The preoperative imaging could guide the surgeon towards the SLN intraoperatively whereas the postoperative imaging may help the pathologist to identify additional potential SLNs to perform additional serial slicing and immunohistochemistry. Based on our results we recommend one preoperative lymphoscintigraphy scan approximately 24 h after tracer injection since the majority of the SLNs were found at the second PET/CT scan (85%) and only three additional SLNs were found at the third PET/CT scan. When the only aim of SLN mapping in colon cancer is improvement of lymph node staging, the method presented here would not hamper introduction of the technique. For SLN biopsy combined with local excision of the primary tumour, this lack of a highly sensitive intraoperative SLN identification technique is a serious drawback To improve intraoperative SLN detection, intraoperative detection using a handheld PET-probe would be desirable. Unfortunately, development of such PET-probes is expensive and quite challenging due to high-energy photons that need a large collimated and shielded detector [19, 28]. Following lymph flow drainage patterns in real-time using NIR-imaging would also be an attractive option to facilitate intraoperative detection. However, in the present study we could not identify fluorescent lymphatic drainage patterns. This is probably the result of the limited penetration depth of NIR-light hampering identification of fluorescent structures beneath a thick layer of fatty mesocolonic tissue.

Besides the limited tissue penetration of NIR-light we also noticed the differences in lymph node identification between NIR and PET/CT-imaging. Firstly, six SLNs located near the primary tumour were not identified using NIR-imaging. This failure is probably the result of tissue overlying the nodes and background fluorescence from the primary tumour. These results suggest that PET/CT is a more reliable imaging modality for SLN mapping in colon cancer. However we must emphasize that the number of included patients is small and NIR-imaging has shown to be a valuable tool to identify SLNs near the primary tumour in several other types of cancer. Another six intraoperatively identified lymph nodes revealed to be fluorescent only. Although these nodes were not located near the primary tumour nor contained metastases, it is uncertain whether these lymph nodes are true non-SLNs. In the currently used method ICG and [^89^Zr]Zr-nanocoll were injected separately, which could have caused different lymphatic drainage patterns of each tracer. Thereby, a considerably higher number of fluorescent-stained lymph nodes were found compared to SLNs identified at PET/CT imaging. This is the consequence of the small hydrodynamic diameter of ICG resulting in fast migration to higher echelon lymph nodes, especially when the time-interval between injection and SLN identification expands.

To overcome these problems we recommend a single simultaneous injection of ICG combined with a radiocolloid. Several studies have shown promising results in multiple types of cancer using the hybrid tracer ICG-^99m^Tc-Nanocolloid, which allows for preoperative SPECT/CT lymphoscintigraphy combined with intraoperative NIR-imaging and gamma-probe guided SLN detection [29, 30]. An advantage of ICG-^99m^Tc-Nanocolloid is the high availability in several countries whereas [^89^Zr]Zr-Nanocoll has not been FDA approved yet. The applicability of this technique in colon cancer should be reinvestigated using knowledge derived from the results presented here on drainage patterns and tracer characteristics.

There were foci we classified as potential SLN at PET/CT imaging preoperatively which were not found in the specimen (see Table 2). Preoperative SLN identification was difficult in several cases due to background scattering. During reassessment of the PET/CT images we noticed that the majority of these foci were in retrospect suspected to be located in the lumen of the colon, so that some radioactive stool (due to leakage of [^89^Zr]Zr-Nanocoll from the injection spot) had been considered as SLN.

For quantification of radioactivity uptake, we calculated the uptake of [^89^Zr]Zr-Nanocoll as a percentage of the injection dose per node using the gamma well counter. Due to logistical restrictions we were not able to weigh nodes and therefore could not express uptake levels as injected dose per gram node (%ID/g). However, the SLNs contained the highest amount of radioactivity compared to the other nodes, indicating that while additional analyses using %ID/g might have provided more information concerning biodistribution of [^89^Zr]Zr-Nanocoll, it would not have changed the results of assigned SLNs.

An important limitation of the present study was the 20% failure rate of [^89^Zr]Zr-Nanocoll injection. Correct needle placement and careful administration of tracer with limited spillage of dye is crucial for a successful SLN procedure. However, submucosal injection is difficult and even more challenging preoperatively when the patient is awake and exposed to the discomfort of a colonoscopy. For accurate tracer injection and image analysis there appears to be a steep learning curve. We therefore advocate that tracer injection should only be performed by an experienced surgeon or gastroenterologist and image analysis by a senior nuclear medicine consultant.

Other disadvantages of the presented study were the limited number of patients, the extensive research protocol and high costs of PET/CT imaging devices. To better determine clinical implications a simplified protocol study including more patients should be performed.

## Conclusion

Perioperative PET/CT lymphoscintigraphy using [^89^Zr]Zr-Nanocoll provides useful anatomical localization information on SLNs in colon cancer and is able to detect nodes near the primary tumour. Use in combination with real-time staining of the SLN is crucial to intraoperatively identify the nodes, since lymph nodes are not visible with conventional white light imaging. The limited penetration depth, the low sensitivity in detecting SLNs near the tumour, and its fast biodistribution are serious drawbacks of ICG for the SLN mapping technique in colon cancer. As a consequence, pre- and postoperative PET/CT lymphoscintigraphy is essential to disclose SLNs to the surgeon and pathologist, respectively. Further research should focus on simplification of the technique presented here, and evaluation of sensitivity rates before SLN mapping can be integrated in the daily treatment of patients with colon cancer. For tracer administration we recommend a single submucosal injection using a composed tracer consisting of a radiocolloid and optical dye. Perioperative SLN identification should consist of a preoperative lymphoscintigraphy scan just before surgery, approximately 24 h after tracer injection. We suggest it should be combined with a postoperative scan of the specimen and intraoperative NIR imaging to identify the preoperatively assigned SLNs.

## Electronic supplementary material

ESM 1 (PDF 12 kb)
