# Supplementary material for: Perioperative PET/CT lymphoscintigraphy and fluorescent real-time imaging for sentinel lymph node mapping in early staged colon cancer
Source: Eur J Nucl Med Mol Imaging. 2019 Feb 23;46(7):1495–505. doi: 10.1007/s00259-019-04284-w (PMC6533411; doi:10.1007/s00259-019-04284-w)
Supplement: Supplementary file 1 — (PDF 12 kb) [file 259_2019_4284_MOESM1_ESM.pdf]

SUPPLEMENTARY MATERIAL 1

TITLE: Perioperative PET/CT lymphoscintigraphy and fluorescent real-time imaging for sentinel lymph node mapping in early staged colon cancer

JOURNAL: European Journal of Nuclear Medicine and Molecular Imaging

M. Ankersmit[1], O.S. Hoekstra[2], A. van Lingen[2], E. Bloemena[3], M.A.J.M. Jacobs[4], D.J. Vugts[2],
H.J. Bonjer[1] , G.A.M.S van Dongen[3], W.J.H.J. Meijerink[5]

[1] Amsterdam UMC, Vrije Universiteit Amsterdam, department of Surgery, Cancer Centre Amsterdam, Amsterdam, The Netherlands

[2] Amsterdam UMC, Vrije Universiteit Amsterdam, department of Radiology & Nuclear Medicine, Amsterdam, The Netherlands

[3] Amsterdam UMC, Vrije Universiteit Amsterdam, department of Pathology, Amsterdam, The Netherlands

[4] Amsterdam UMC, Vrije Universiteit Amsterdam, department of Gastroenterology, Amsterdam, The Netherlands

[5] Department of Operation Rooms and MITeC Technology Center, Radboud University Medical Centre, Nijmegen, The Netherlands

Corresponding author:
M. Ankersmit
m.ankersmit1@vumc.nl

Detailed describtion of the injection technique

Colonic lavage occurred the day before the first colonoscopy with 2L Moviprep©.

During first colonoscopy the PET-tracer [^{89}Zr]Zr-Nanocoll was injected using a V960 injection needle with a luminal volume of 1.2 mL (Prince Medical, Gutenberg, France). After endoscopic tumour localization correct submucosal needle placement was confirmed by a raising bleb of NaCl 0.9% directly followed by injection of [^{89}Zr]Zr-Nanocoll and final flushing with 2.0 mL NaCl 0.9% to achieve maximal tracer administration. Afterwards the injection needle was measured for radioactivity to establish the exact amount of injected dose of [^{89}Zr]Zr-Nanocoll.

The second colonoscopy was performed directly after general anaesthesia and laparoscopic port placement. After localization of the tumour, a laparoscopic clamp was placed 5-10 cm proximal to the tumour to prevent distension of the proximal bowels. Injection of ICG/NaCl solution (2.5 mg/mL) was performed by the same senior gastroenterologist, again using a V960 injection needle. ICG/NaCl was administrated by a single shot at the base of the tumour similar to injection of [^{89}Zr]Zr-Nanocoll. After confirmation of correct needle placement in the colonic submucosa using NaCl 0.9%, 0.5 mL (0.2-2.0 ml) ICG/NaCl was administered.
